# Malignant Pleural Effusion in Pediatrics: A Rare Presentation

**DOI:** 10.7759/cureus.33283

**Published:** 2023-01-02

**Authors:** Lubna Almogarry, Alzahra Y Alradhi, Abdullah Alshamrani

**Affiliations:** 1 College of Medicine, Imam Abdulrahman Bin Faisal University, Dammam, SAU; 2 Department of Pediatrics, King Fahad Medical City, Riyadh, SAU; 3 Department of Pediatrics, Alfaisal University College of Medicine, Riyadh, SAU

**Keywords:** parapneumonic effusion, lymphoma, malignancy, effusion, pneumonia

## Abstract

Pleural effusion is the most common presentation of pleural diseases. It is relatively common in children with two predominant types: exudative and transudative effusions. In children, exudative types are the most common with bacterial infection being the most prevalent cause. In some cases, effusion could be difficult to confirm.

We describe two patients with a similar age group who presented with respiratory distress in the form of fever, cough, and shortness of breath. They were managed clinically and radiologically as cases of parapneumonic effusion. Both were started on antibiotics with no improvement. After reviewing the cases, it was discovered that some crucial aspects of the history and physical examination that were essential to reach the correct diagnosis had not been taken into consideration.

Parapneumonic effusion should be taken with caution, meticulous history and examination are warranted, and lymphocytic-predominant effusion is very alarming for potential malignancy in the absence of tuberculosis infection. If the antibiotic medication yields no significant improvement, earlier referral should be considered.

## Introduction

Pleural effusions can be caused by a variety of conditions. These include pleuropulmonary pathology or systemic disorders. Pleural effusions are classified into two categories: transudates and exudates. Transudates have narrow differential diagnoses, with the most common being congestive heart failure and hypoproteinemia, whereas exudates have several causes [1]. Parapneumonic effusion/empyema is the most prevalent cause of effusion in children. Malignant effusions, on the other hand, are rarely reported in the pediatric literature. Non-Hodgkin lymphoma accounts for half of the cases, with neuroblastoma, Ewing's sarcoma, rhabdomyosarcoma, and Wilm's tumor accounting for the minority of cases [1]. Small pleural effusions could be asymptomatic, and when they get larger to compress the lung tissue, dyspnea, tachypnea, and chest pain may develop [2]. Exudates usually have high specific gravity, high protein (>3 g/dL), high lactate dehydrogenase (LDH) levels, and high white blood cell (WBC) count. Moreover, they present with low pH (<7.2) and glucose levels (<40 mg/dL) [2]. Chest radiographs are used for confirming the presence of effusions and ultrasound or chest computerized tomography (CT) scan is also used for detecting and distinguishing pleural fluid from parenchymal lesions and pleural masses [3]. In this paper, we present two cases diagnosed initially as parapneumonic effusion with variable responses to the initial management. The first case was masked with starting steroid treatment as the patient is known to have asthma. However, upon discharge, the condition relapsed. On the other hand, the second case had enough duration of antibiotics with a significant response.

## Case presentation

Case 1

A seven-year-old Saudi boy, a known case of asthma, presented to the emergency department (ED) with a history of fever, cough, and shortness of breath for three days. He also complained of mild chest pain, which was right-sided, worsened with inspiration, and resolved with paracetamol. His parents also noted weight loss for two weeks and lethargy. On examination, the patient looked unwell, moderately dehydrated, tachypneic, and in respiratory distress. His vitals showed a temperature of 38.6 degrees Celsius (°C), a heart rate of 159 beats per minute (BPM), blood pressure of 109/60 millimeters of mercury (mmHg), respiratory rate of 38 breaths per minute, oxygen saturation of 90% on room air and 96% on nasal cannula of 1 liter per minute (LPM). The patient had a mild congested throat, and there was no lymphadenopathy on examination or clubbing. His growth parameters fall within the normal range. On chest examination, the patient was noted to have chest expansion asymmetry, with marked reduced breath sounds on the right side and scattered crackles, and no bronchial breathing was heard. The patient also had dullness on percussion on the right side. Systemic examination was unremarkable.

**Figure 1:**
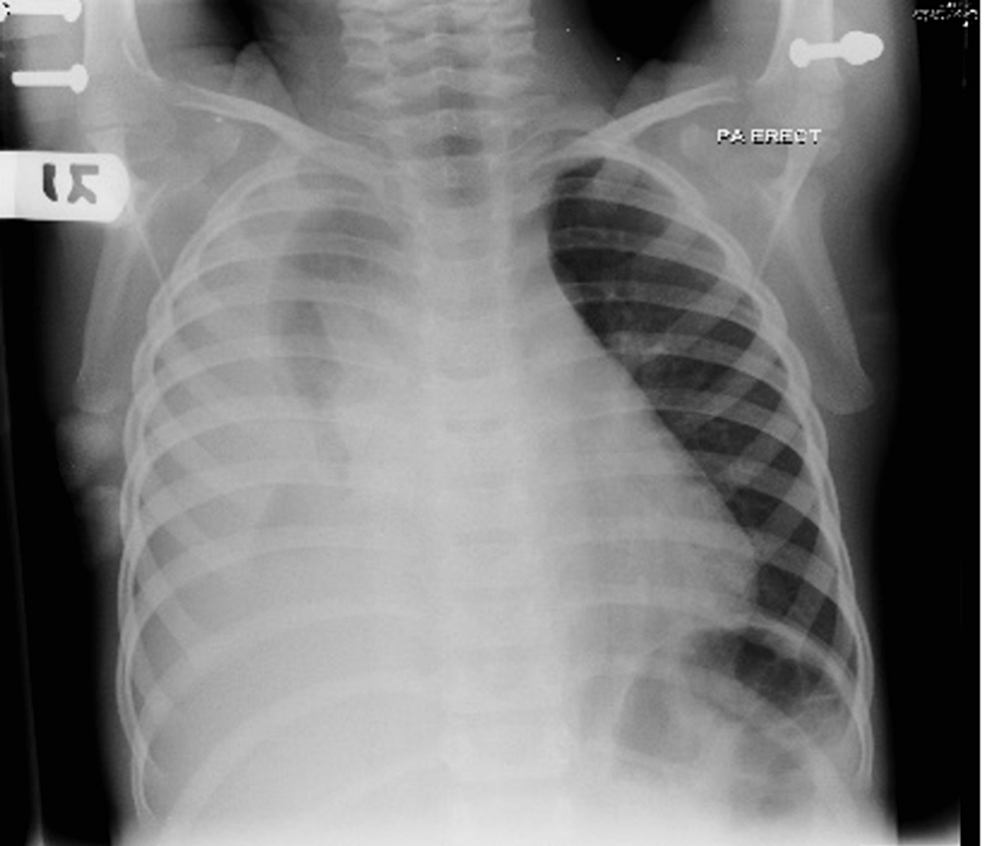
Plain chest X-ray, posteroanterior view, showing a diffuse opacity occupying most of the right-sided chest obscuring the costophrenic and the cardio-phrenic angle, a tracheal deviation to the left side with absence of air bronchogram, and positive meniscus sign.

**Figure 2:**
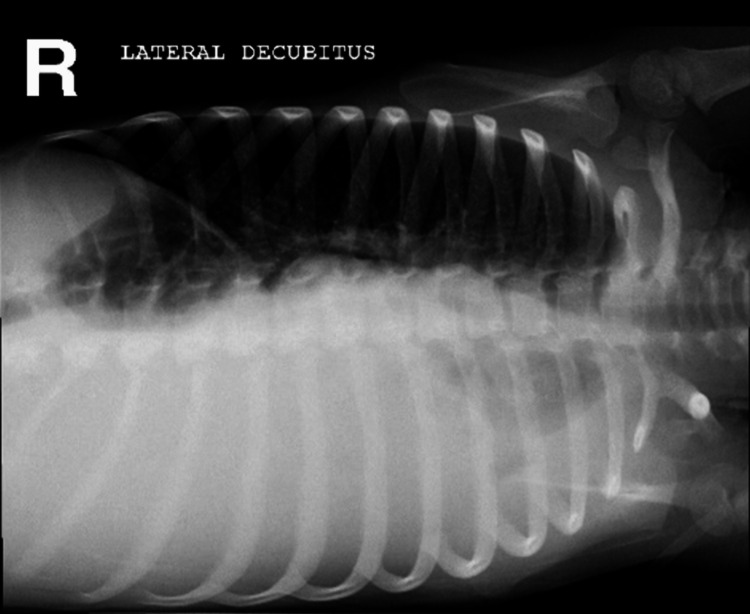
Plain chest X-ray, right lateral decubitus view, showing a large free fluid level occupying more than 50% of the ipsilateral hemithorax.

Initial investigations revealed a normal complete blood count (CBC), with an erythrocyte sedimentation rate (ESR) of 70 millimeters per hour (mm/h). The initial chest X-ray showed a diffuse opacity occupying most of the right-sided chest obscuring the costo-phrenic and the cardio-phrenic angles. A tracheal deviation to the left side with the absence of an air bronchogram and positive meniscus sign was also noted (Figure 1). Prior findings were highly suggestive of a large right-sided pleural effusion and were further confirmed in the right lateral decubitus view that showed a large free fluid level occupying more than 50% of the ipsilateral hemithorax (Figure 2). This patient was initially admitted to the general pediatric ward as a case of asthma exacerbation with parapneumonic effusion. Treatment was started with cefotaxime and clindamycin along with bronchodilators and steroids.

**Figure 3:**
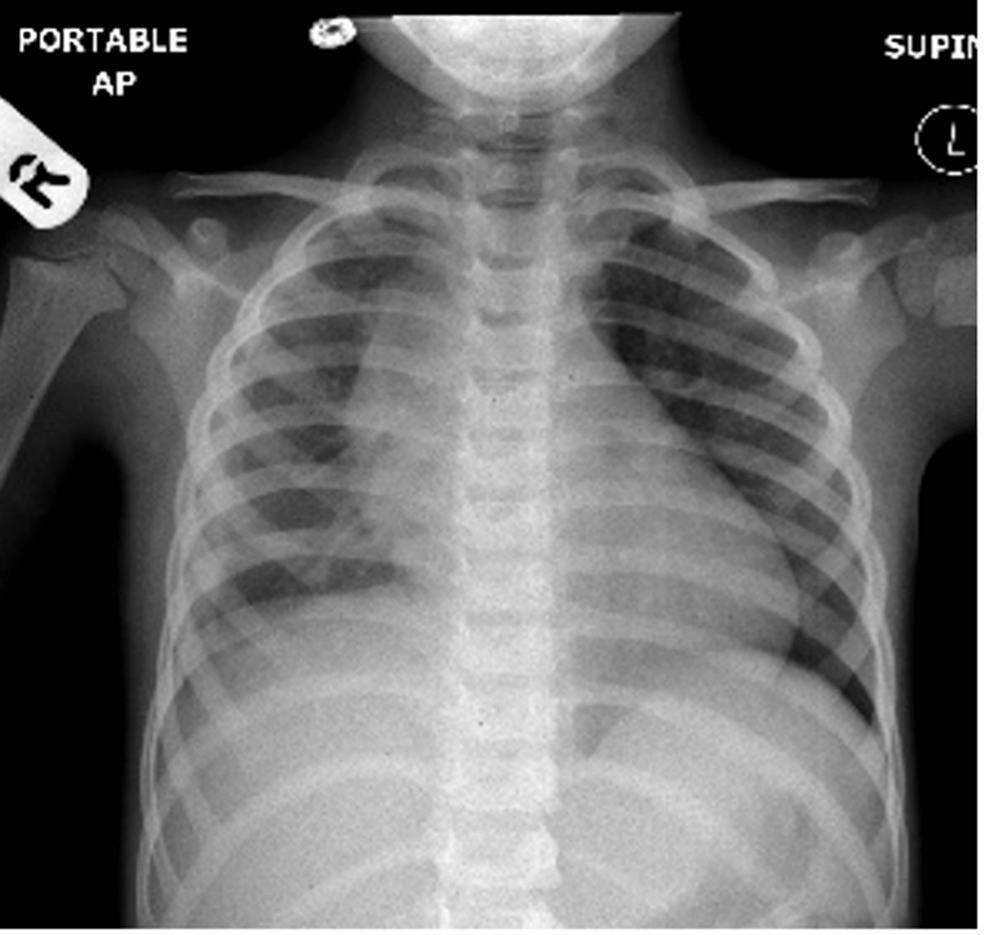
Plain chest X-ray, anteroposterior view, showing a marked resolution of the effusion, thick pleural surface, reduced lung volume on the right side, and potential widened mediastinum.

Since the effusion was significant, a chest tube was inserted and pleural fluid analysis showed a high LDH ratio of 1563:871, a high protein level of 35 milligrams per deciliter (mg/dL), glucose 3.1 millimoles per liter (mmol/L), and increased WBC count reported initially as 80% neutrophils. Surprisingly, the patient showed marked improvement and he was discharged on the third day after the fever resolved with marked improvement on chest X-ray (Figure 3). The patient was sent home on double antibiotic therapy with a follow-up plan.

**Figure 4:**
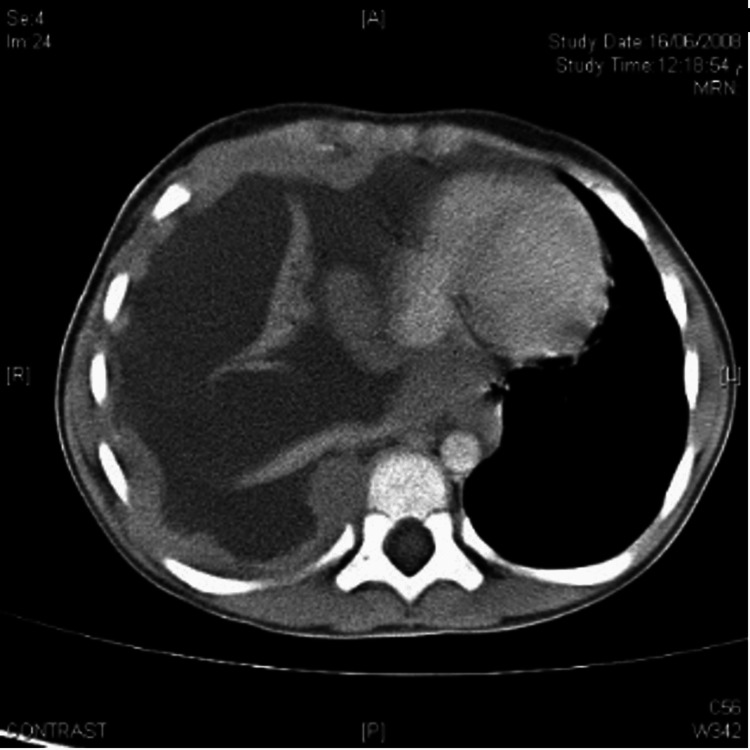
Computed tomography (CT) scan, axial view, mediastinal lung window shows a marked right-sided pleural effusion, collapsed lung, thick pleural service, shift of mediastinum to the left side.

**Figure 5:**
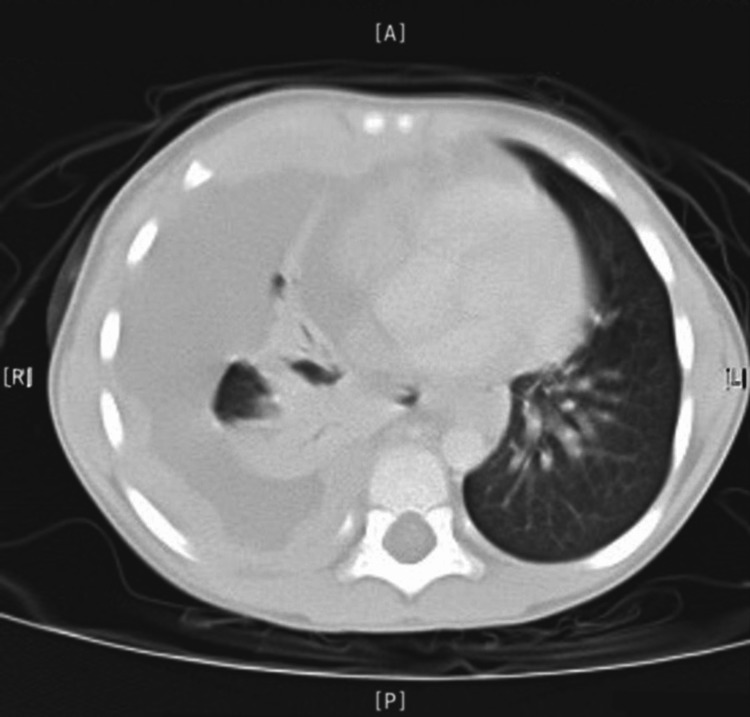
Computed tomography (CT) scan, axial view, lung window shows a marked right-sided pleural effusion, collapsed lung, thick pleural service, shift of mediastinum to the left side.

He was re-admitted within 48 hours with a similar presentation of high-grade fever and similar chest findings. Upon the second admission, further investigations were done. A chest CT scan was done and revealed a large right-sided pleural fluid, collapsed right lung, nodular pleural surface, and a potential mediastinal mass (Figures 4, 5).

**Figure 6:**
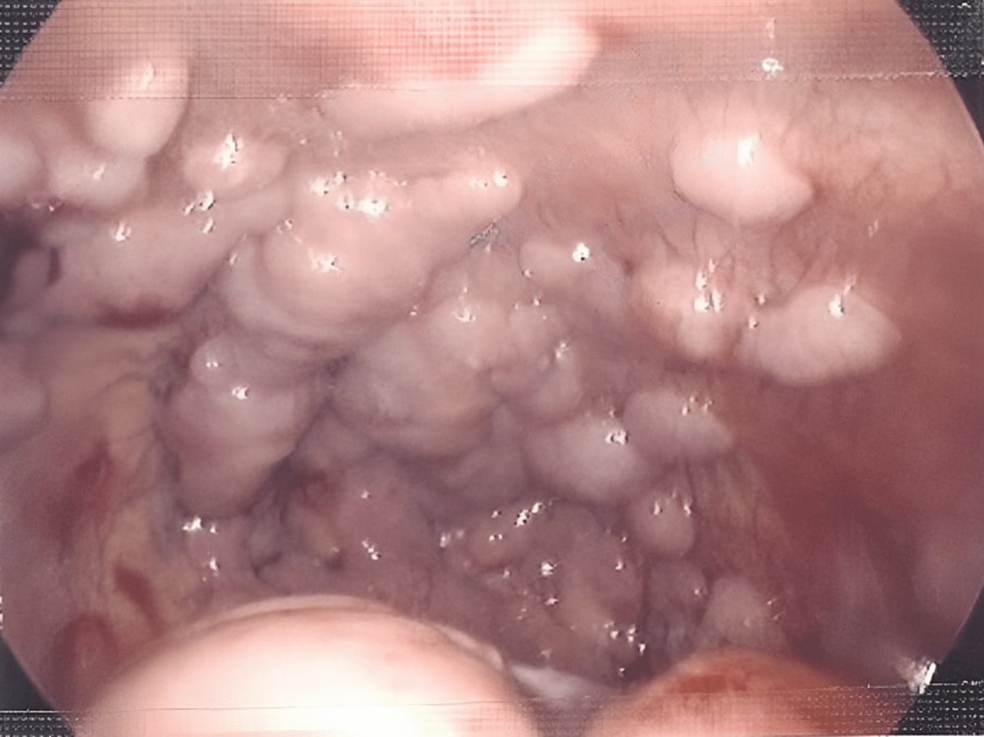
Thoracoscopy showing multiple nodular pleural surface.

The culture of pleural fluids was negative. A thoracoscopy was scheduled for inspection and decortication, and a multiple nodular pleural surface was very suggestive of malignant origin (Figure 6). Revised history revealed poor appetite and decreased activity for several weeks, and missed hepatomegaly in the abdominal examination which was confirmed by ultrasound to be 4 cm below the costal margin. A multidisciplinary team was involved and the diagnosis was confirmed by thoracoscopy in which the nodular pleural as well as right cervical lymph node were taken for cytology and came back to be B-cell Hodgkin's lymphoma-stage IV and the patient was referred to oncology.

 Case 2

A six-year-old Saudi boy was admitted to the general pediatric ward with a two-week history of cough and fever that did not respond to oral amoxicillin/clavulanic acid. Four weeks prior to the ED visit, the child was observed to have a poor appetite and reduced activity, and his condition got worse as his fever reached 40°C and he developed a progressive cough; for this, he was evaluated in the primary care clinic and diagnosed clinically as bronchopneumonia and prescribed oral amoxicillin/clavulanic acid 457 mg, 6 mL twice a day with paracetamol. Unfortunately, he did not respond to the ten-day management course as his cough became wet. Further, he became more tachypneic with left-sided chest pain. The patient lost 2 kg over this period.

On examination, the patient looked ill, his vital signs showed a temperature of 39°C, a heart rate of 140 BPM, blood pressure of 110/65 mmHg, and oxygen saturation of 87% on room air but 99% on nasal cannula of 1 LPM. When auscultating his chest, there were marked reduced breath sounds on the left side, left-sided stony dullness on percussion, and no added sounds. Systemic examination showed mild hepatosplenomegaly, otherwise normal.

**Figure 7:**
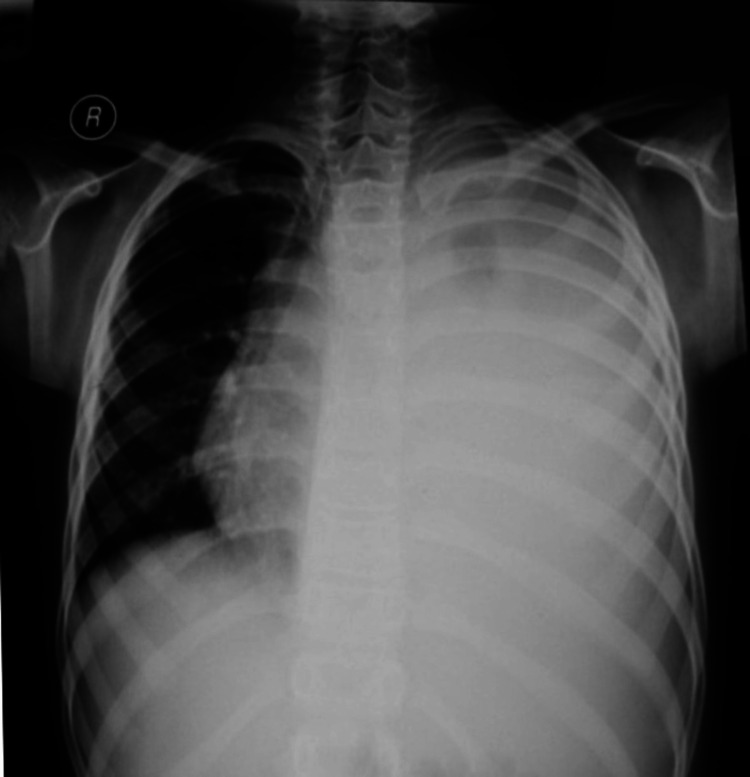
Plain chest X-ray, posteroanterior view, showing a diffuse opacity occupying the right hemithorax, with more homogeneous opacity in the middle and the lower zones, obscuring the cardiac, costophrenic, and the costo-diaphragmatic angle, and ascent of air bronchogram, trachea deviated to the right side consistent with large left-sided pleural effusion.

The patient was admitted initially as a case of complicated pneumonia (left-sided parapneumonic effusion). Chest X-ray was done on admission that showed a diffuse opacity occupying the left hemithorax, with more homogeneous opacity in the middle and the lower zone, obscuring the cardiac, costo-phrenic, and costo-diaphragmatic angles. There was also some pressure effect on the mediastinum, especially at the tracheal level. Furthermore, increased right intercostal space was noted; however, there was an absent air bronchogram (Figure 7). Prior findings were highly suggestive of large left-sided effusion. Mild secondary hyperinflation on the right side was also noted supporting the provisional diagnosis.

Chest CT was requested to further investigate, confirming a large pleural effusion, collapsed left lung, and a very large chest mass compressing the left main bronchus. Blood and pleural fluids were sent for investigation. Blood results are shown in Table 1; pleural fluid was hemorrhagic, with negative gram stain, WBC 1318 per microliter, predominant lymphocyte 91%, and LDH 1219 units per liter. Bone marrow confirmed the malignant cells and the patient was referred to oncology as lymphoma-stage III.

**Table 1:** Blood work-up results during the patient's second admission.

Lab value	Result
White blood cell (×10^9^/L)	18.900
Hemoglobin (g/dL)	9.0
Platelet count (×10^9^/L)	648.000
Prothrombin time (seconds)	16.1
Partial thromboplastin time (seconds)	43
International normalized ratio	1.4
Fibrinogen (g/L)	3.8
Ferritin (µg/L)	64
Erythrocyte sedimentation rate (mm/h)	98
C-reactive protein (mg/L)	138
Procalcitonin (ng/mL)	0.36
Uric acid (mg/dL)	10
Serum lactate dehydrogenase (u/L)	590

## Discussion

We reported two cases of patients presenting with fever, cough, and shortness of breath. Both patients were further investigated, and a chest X-ray revealed a picture of significant pleural effusion. Our first case was challenging as there were three key points affecting the patient's hospital course. The first was missing hepatomegaly in the examination. The second occurred when a lab error in the examination of pleural fluid reported a high WBC count with 80% neutrophils. Thus, the decision was to keep the patient on double antibiotics as the patient was improving. The third point was starting the patient on steroids as he was known to have asthma. Steroids played a role in the patient's response to the initial management; as soon as the steroids were stopped, the patient's illness relapsed. Chest CT was performed in both cases as it was extremely essential in determining the proper diagnosis. This case highlights the importance of considering a broad differential diagnosis in children presenting with a picture of pleural effusion since it may be the initial manifestation of malignancy. The pleural cavity is a potential space created within the two opposing pleurae overlying the lungs. This narrow space normally contains a few milliliters of pleural fluid [4]. The pleural fluid is not only produced by the parietal pleura but also resorbed essentially by the lymphatic vessels lying in the parietal pleura. A pleural effusion will only be noted when fluid production exceeds the lymphatic vessels’ ability to resorb, due to an increase in production, a decrease in resorption, or a mix of the two [5]. Pleural effusions in children commonly are of infectious cause (usually parapneumonic) ranging from 50% to 70%, and to a lesser frequency congestive heart failure 5-15%, and rarely malignancy [1]. Malignant pleural effusion (MPE) can be described as the collection of a large amount of exudate within the pleural space, associated with the presence of malignant cells or tumor tissue [6]. It is also worth mentioning that the most prevalent MPE cause in children is non-Hodgkin's lymphoma, unlike our first case in which our patient was eventually diagnosed to have Hodgkin's lymphoma [2]. The pathophysiology of MPE is thought of as a result of a direct and indirect relation with the malignancy. The direct causes involve direct tumor involvement and lymphatic system disruption, whereas an indirect cause includes increased capillary permeability as a result of local inflammatory changes due to tumor invasion [3]. The clinical presentation of MPE in pediatrics heavily relies on the primary cause. However, they usually present because of generalized weakness, malaise, fever, weight loss, dyspnea, and chest pain. In other instances, patients are asymptomatic and only present with low-grade fever and cough [7]. It is also worth noting here that the first case, which was diagnosed as Hodgkin's lymphoma, presented solely because of a cough and fever that did not resolve spontaneously in three days, whereas the second case, diagnosed as lymphoma, came in as a second presentation due to failure of antibiotics. Moreover, another case report of an eight-year-old with a primary rhabdomyosarcoma of the pleura also presented with a progressive dry and painful cough for eight weeks [8]. A general examination of a child with MPE might show mild-to-moderate respiratory distress, cachexia, dyspnea, and anxiety due to pain, hypoxemia, or discomfort [9]. On auscultation, pleuritic chest rub might be heard in the early stages that decreases as the pleural fluid accumulation increases [2]. With time and the increase of pleural fluid accumulation, mediastinal shift and displacement of the cardiac apex as well as the trachea to the contralateral side might be observed. Large fluid collection causes fullness in the intercostal space and diminished chest excursion on the affected side [7]. A missed systemic examination initially in both of our cases, which eventually showed hepatomegaly, could be of clinical importance. However, a similar case report mentioned that there was neither lymphadenopathy nor hepatosplenomegaly during systemic examination [8]. One of the primary investigations that are done when suspecting pleural effusion is a chest X-ray. It can aid in the confirmation of the location and size of the pleural effusion [2]. Another initial investigation is chest ultrasonography, which has a 100% sensitivity in diagnosing pleural effusion [3]. Thickened pleural wall (greater than 10 mm), thickened diaphragmatic wall (greater than 7 mm), and nodularity in the chest ultrasound are highly suggestive of malignancy. Chest CT is superior to the first two as it visualizes in great detail the lung parenchyma, pleural surfaces, mediastinum, and chest wall [2]. However, about 50% of MPE patients do not have any pleural abnormalities on CT. Diagnostic thoracentesis should be done for every patient that has more than 10 mm of free pleural fluid on chest X-ray, ultrasound, or CT. MPE's gross appearance can range from bloody to cloudy or even clear. Moreover, bloody pleural effusions are most commonly caused by malignancy, and around half of MPEs are bloody. Pleural fluid analysis is undertaken to differentiate between exudative and transudative effusions. This is done by following Light’s criteria [3]. Exudative pleural effusion meets one or more of the following conditions: (1) the ratio of pleural fluid protein to serum protein exceeds 0.5, (2) the ratio of the pleural fluid LDH to serum LDH exceeds 0.6, and (3) the LDH pleural fluid is more than two-thirds the upper normal limit for serum LDH [2]. Common exudative causes include infection, malignancy, gastrointestinal disease, and pulmonary embolism, whereas transudative causes include heart failure, renal failure, and cirrhosis. Furthermore, Light’s criteria have 100% sensitivity in exudate diagnosis; however, it has a 25% false positive rate [3]. Pleural fluid cytology is used for definitive diagnosis. MPE generally shows lymphocyte-predominant fluid [3]. Other lymphocyte-predominant pleural fluid causes are tuberculosis (TB), para-pneumonic, chronic inflammatory, sarcoidosis, rheumatoid pleuritis, and chylothorax [3,10,11]. The diagnosis of MPE can be surely done by the demonstration of malignant cells in the pleural fluid sample [3]. In around 50% of MPE cases, malignant cells are demonstrated in the pleural fluid [2]. A highly suspicious case, mentioned in another report, did pleural effusion exfoliative cytology as an initial test and showed large lymphoid cells that greatly aid in the diagnosis of plasmablastic lymphoma [12]. The management of MPE must be in a multidisciplinary approach. Thoracocentesis is both diagnostic and therapeutic, as it relieves symptomatic MPE. Tube thoracostomy can be utilized in patients with recurrent MPE and a short life expectancy [2]. It is of high importance to have coordinated care and to stage patients as quickly as possible to receive appropriate management.

## Conclusions

Pleural effusion is common among the pediatric age group. In this report, we presented two malignant effusion cases that had an intricate hospital course. Reviewing the condition in a multidisciplinary approach, better communication within healthcare specialists, and proper history-taking could have accelerated their trip to their appropriate management.
